# Supplementary material for: Patterns of Intron Gain and Loss in Fungi
Source: PLoS Biol. 2004 Nov 30;2(12):e422. doi: 10.1371/journal.pbio.0020422 (PMC532390; doi:10.1371/journal.pbio.0020422)
Supplement: Table S1 — Also available at http://genes.mit.edu/NielsenEtAl/. (4.3 MB ZIP). [file pbio.0020422.st001.zip › NielsenEtAl/html/655.html]

AN6855.1.NCU03597.1.MG00706.1.FG07021.1


```
 CLUSTAL W (1.82) Multiple Sequence Alignments - Introns Inserted


Sequence 1: NCU03597.1	667 aa
Sequence 2: MG00706.1	666 aa
Sequence 3: FG07021.1	655 aa
Sequence 4: AN6855.1	905 aa
Alignment Length: 937 aa
Number Identitical Residues: 257 aa
Alignment Score (without introns) 14158


MG00706.1 	------------------------------------------------------------
NCU03597.1	------------------------------------------------------------
FG07021.1 	------------------------------------------------------------
AN6855.1  	MDIPSIDPSYLKLFQAVESRFAQATSIPADKWAILATTTLAAGPDPERADQLYLYLTSQP
          	 . .: ..:  .  .: .:  :.::: .:.. :  :::: ::..... :..     ::..

MG00706.1 	------------------------------------------------------------
NCU03597.1	------------------------------------------------------------
FG07021.1 	------------------------------------------------------------
AN6855.1  	AYSTSDARKQLVRRLREALLKSVIIVGVCKPIEAILAISKVEKEEDKDYSSATREGWACD
          	: :::.: ..      .:  .:    . ... .:  : :. ....... :::: .. :..

MG00706.1 	------------------------------------------------------------
NCU03597.1	------------------------------------------------------------
FG07021.1 	------------------------------------------------------------
AN6855.1  	EANHERGVTWFNKLYARNGNATLDLFAAHKDFSWLSTEITYGLFLSDRQVLDDVDTQMVV
          	.:. . . :  ..  : ...:: .  :: .. :  ::. : .   :. .  .. .:.   

MG00706.1 	--------------------------------------------------~---~-----
NCU03597.1	--------------------------------------------------~---~-----
FG07021.1 	--------------------------------------------------~---~-----
AN6855.1  	LPGIMSQNLPNETHWHIRGTRRLGVPIEDVKIVTECVKIVADFYGNKLDK0GDC1LEATN
          	 ..  :.. ...:     .:   . . .. .  :.. .  :.  ... .. ...  .::.

MG00706.1 	--------------MADPIVEELNERFAPGGKP-LEVDVMGELQSIMRLHLLSPQDLFFK
NCU03597.1	--------------MADPVVAELNERFGTANTE-LSPDVIYELQSIMRLHELDVQDMFFK
FG07021.1 	--------------MAD---AEIQDRFSP-NKP-LEPDVLSELESIMRLHGLSAEDLFFK
AN6855.1  	LAEMGSILSDDPGAAADATKDELTELFAPSAPSGLPADILMELQSIMRVHSITAQELFYR
          	 :. .:  :....: **.   *: : *...   .*  *:: **:****:* :  :::*::

MG00706.1 	WESYCIKMDMDEMRPKYDTIRGFKQDLQDALERNNRSQA-AHVKTEKRMGATPRAGGKGA
NCU03597.1	WESYCIKMDMDQPKMSMSTVRKFKQDLQDTLERANRAQALEQVKTEKRPGGTPRPGARNN
FG07021.1 	WESYCIKLDIDAQVLSLEALRNLKQSIQDELEN---SHRKVQVKSERKVASAPRGG--NK
AN6855.1  	WESYCLRMGSEETKLDLETVRLFKRDVQENLER--QARGRQQVEKRGGAAATPKARDMPV
          	*****:::. :    . .::* :*:.:*: **.   ::   :*:..   ..:*:      

MG00706.1 	GG-----DVFGM2LDGLVPS-TPGTS-KLNK--SGSARRKNMETPSMSRVKSEHPASSPS
NCU03597.1	S------DVFGM~LDGLT---TPAAR-RAAK--LGSARKTPMGTPIGSRLKNE-PVSSPM
FG07021.1 	GG-----DVFGM~LDGLVPS-TPASGSKLNRGAGGSAIKKKLDTPKG-------LTSSPA
AN6855.1  	GGGTVAADVFGM2LDGLTPARTPGAG--------SAKRKAEFSSPSVAK-----KTNSPL
          	...: ::***** ****..: **.:         .:  :  : :*  :       ..** 

MG00706.1 	T-LERQLDSLG-ALT2PSSFNDRANPGEIIEVLNNQLPAAEPPIAPFSESRIKLTAASDL
NCU03597.1	K-LEAQLDAMG-VIP2PSSFKDRPNAGEVIEILNENLPAPEPPIAPFGESRIKLTAASDL
FG07021.1 	KGMNDQLKSLN-NLQ2PTSFGDRTNPGETIEILNDSLNAAEPPIAPFSEPRVKLTAASDQ
AN6855.1  	ASKTNVANGNGEGLQ2SVPFSERQNPGQTIETINEHLSMPETPMAPPSEPRIRPAANTDL
          	 .     .. .. :  . .* :* *.*: ** :*: *  .*.*:** .*.*:: :* :* 

MG00706.1 	KKLGYRPMAMKLSEASEILDDRIEEFTNFVMQHHNLDESAFGSAANQSTSEIICVGRIAS
NCU03597.1	KKLGYKPLAMKLSEASEILDDRIDDFTSLVLEHYKLDESVFGSAAMQSTTEIVAVGRVAS
FG07021.1 	KKLGYKPLAMKLSEASEILDDRIDNFMALVQQHHKIDESEFGSAAAQSTTEVVAVGRIAS
AN6855.1  	KKFGYKPMAMRLSEASEILDDRIDEFMTIFEKMYEKEDVTFGSAAVQSTNEIVAVGRIAS
          	**:**:*:**:************::*  :. : :: ::  ***** ***.*::.***:**

MG00706.1 	DTSEGRLNAASLVLETSRRSGGGLRAPLNLQKLPGYQFFPGQIVALRGTNASGHEFNVSE
NCU03597.1	DTSEGKLNAASLVLETSRKLGGGFRVPLNVSKLKGYQFFPGQIVALRGTNTTGREFTVNE
FG07021.1 	DSPEGKLNAASLVLETSRRTGMGLRVPLKMDSVRSWSFFPGQIVAFRGTNASGSEFVVKE
AN6855.1  	DSPEGKLNPASLVLETSRRSGAGRRVPLNVDSIPSVNFFPGQIVALLGINASGNYFSVKE
          	*:.**:**.*********: * * *.**::..: . .********: * *::*  * *.*

MG00706.1 	VLQIPLLPNAASTPEALEAHRQRLQGGPDAMDSDSD-PMPLNVMFASGPYSADDNLDFEP
NCU03597.1	VIRMPLMGNAASTLETMAAHRERLRGGPDAMDTDDENPMPLNVIFASGPYTADDNLDFEP
FG07021.1 	ILDMPILPSAASSASTLEVHREKFRGGPDAMDEDSE-PAPLNILYASGPYTADDNLDFEP
AN6855.1  	VLPPPLLPPAVSSVPTIESTNERLSEGG---------SSPLNVMIACGPYTADDNLDFEP
          	::  *::  *.*:  ::   .:::  *          . ***:: *.***:*********

MG00706.1 	LHTICNQAADTYADALVLTGPFIDIDHPLIAAGDFDLPEDANIDPDQATMATLFKYMISP
NCU03597.1	LHTFINEAADTYADAVILTGPFIDAEHPLIASGDFDLPEEAVYDPDAATLNTVFKYLISP
FG07021.1 	LHALCSQAADTYADALVLTGPFLDIDHPLIATGDFDLPEEANFDPDTVTMSTVFKYLVAP
AN6855.1  	LKEICHKAAEGYADGLILLGPFLDLEHPLLASGDFELPES--IDPDTATLTTVFKHYIST
          	*: :  :**: ***.::* ***:* :***:*:***:***.   *** .*: *:**: ::.

MG00706.1 	ALNQLAASNPQVTILLIPSVRDAIDKHVSWPQDAFPRKDLGLPKAAKIVGNPMTFSINET
NCU03597.1	ALNRLVSANPHVTILLVPSVRDVIDRHVSWPQDPFPRKELGLHKSIKILGNPMTLSLNET
FG07021.1 	AFSRLASSNPQITIVLVPSVRDVLSKHVSWPQDTIPRKELGLPKAVRIVTNPMTLSINEV
AN6855.1  	PLQQLAAAVPSITIALVPSVRDAVSKHVSWPQEQLPKKELGLPKQARMVSNPVTFSLNET
          	.:.:*.:: * :** *:*****.:.:******: :*:*:*** *  ::: **:*:*:**.

MG00706.1 	LVGISSQDVLYQLQGGVLAG-GKAPDGMDRLSRLARNVIEQRHYFPLFPPMDRSKLPKTG
NCU03597.1	LVGISSQDALWELKHEELIG-GTVED-TNGLSRVARYLIEQRHYFPLFPPTDRTKLPKTG
FG07021.1 	VLGVSSQDILSQLRSEEVVSRGGAQPGGDLMSRLCRYLVEQRHYFPLFPPTERSKLPKTG
AN6855.1  	VIGMCSHDVLYELRREEALH--GKPKEGNLLTRLVKYLIEQRHFLPIFPPSSRDALPRPG
          	::*:.*:* * :*:              : ::*: : ::****::*:*** .*  **:.*

MG00706.1 	---TAEELALGSVLDVSYLKLGEMVNVRPDVLVLPSAMPPFAR0VVESVLIINSGYLSKR
NCU03597.1	RQVIDGQAAPGAMLDISYLKLGEMVNVRPDVLIVPSALPPFAK0VIESVLVINPGYLSQR
FG07021.1 	---TEDGVATGAVLDLSYLELGEMVNVRPDVMVVPSLLPPFAK0VVESVLLINPGYLSKR
AN6855.1  	---FEGGLATGAALDVSYSKLGEWWNVRPDILIVPSMLPPFVK0VVDSILTINPGTLSKR
          	        * *: **:** :***  *****::::** :***.: *::*:* **.* **:*

MG00706.1 	RGPGTYARMTLYPPKVPQ-EGTPHGVVGHSVYERARVEITRI
NCU03597.1	KRAGTYARMTLYPPKLSEADAAPGGMLAHRIFERARVEVTRI
FG07021.1 	RGAGTYARMTLYPPAASG--GSGDAMMSHQVFERARVEIVRI
AN6855.1  	RAAGTFAQMAIHPRVVSE-EEREQKNISHKLYERTRVDIIRI
          	: .**:*:*:::*   .  .      :.* ::**:**:: **
```
